# A Malay version of the Child Oral Impacts on Daily Performances (Child-OIDP) index: assessing validity and reliability

**DOI:** 10.1186/1477-7525-10-63

**Published:** 2012-06-08

**Authors:** Zamros YM Yusof, Nasruddin Jaafar

**Affiliations:** 1 Department of Community Dentistry, Faculty of Dentistry, University of Malaya, Kuala Lumpur, 50603, Malaysia; 2 Community Oral Health Research Group, University of Malaya, Kuala Lumpur, Malaysia

**Keywords:** Oral health, Quality of life, Reliability, Schoolchildren, Validity

## Abstract

**Background:**

The study aimed to develop and test a Malay version of the Child-OIDP index, evaluate its psychometric properties and report on the prevalence of oral impacts on eight daily performances in a sample of 11–12 year old Malaysian schoolchildren.

**Methods:**

The Child-OIDP index was translated from English into Malay. The Malay version was tested for reliability and validity on a non-random sample of 132, 11–12 year old schoolchildren from two urban schools in Kuala Lumpur. Psychometric analysis of the Malay Child-OIDP involved face, content, criterion and construct validity tests as well as internal and test-retest reliability. Non-parametric statistical methods were used to assess relationships between Child-OIDP scores and other subjective outcome measures.

**Results:**

The standardised Cronbach’s alpha was 0.80 and the weighted Kappa was 0.84 (intraclass correlation = 0.79). The index showed significant associations with different subjective measures viz. perceived satisfaction with mouth, perceived needs for dental treatment, perceived oral health status and toothache experience in the previous 3 months (p < 0.05). Two-thirds (66.7%) of the sample had oral impacts affecting one or more performances in the past 3 months. The three most frequently affected performances were cleaning teeth (36.4%), eating foods (34.8%) and maintaining emotional stability (26.5%). In terms of severity of impact, the ability to relax was most severely affected by their oral conditions, followed by ability to socialise and doing schoolwork. Almost three-quarters (74.2%) of schoolchildren with oral impacts had up to three performances affected by their oral conditions.

**Conclusion:**

This study indicated that the Malay Child-OIDP index is a valid and reliable instrument to measure the oral impacts of daily performances in 11–12 year old urban schoolchildren in Malaysia.

## Background

Oral health related quality of life (OHRQoL) instruments were developed to measure subjective oral impacts on daily performances and quality of life [1]. Their use enables important information on the functional and social dimensions of dental diseases and illness to be collected. This information is crucial as it reflects the perceived oral health needs of individuals and how they feel about their oral health and its influence on quality of life. Traditionally, oral health need has been estimated by using professionally based measures. Although such measures are important, they do not take into account the impacts of oral conditions on daily life and well-being when estimating oral health needs [2]. For example, a mild oral condition, such as malocclusion, may not require treatment when measured using a clinical measure but may have a wider repercussions on the person’s ability to talk, smile and socialise [3]. Thus, OHRQoL instruments play a central role alongside clinical measures for estimating oral health needs and status of individuals.

The Child Oral Impacts on Daily Performances (Child-OIDP) is one of the most widely used OHRQoL instruments to be used in children [4]. It was developed in 2004 and was successfully tested on 11–12 year old Thai schoolchildren on the prevalence and severity of oral impacts on daily activities [5]. The Child-OIDP derived its theoretical framework based on modifications from the WHO’s International Classification of Impairment, Disabilities and Handicaps [1,6]. As such, it allows measurement of a range of OHRQoL dimensions including oral health impairments, functional limitations and disability [7-9], thus making it suitable to measure oral impacts in children. The Child-OIDP index measures oral impacts on eight performances i.e. eating, speaking, cleaning teeth, relaxing, emotional stability, smiling, doing schoolwork and socialising. It is relatively short, comprehensive and easy to use. Its scoring system directly relates perceived oral health needs with the severity of impact scores. Since its use among the Thai schoolchildren, the Child-OIDP index has successfully been translated and tested in various other settings to estimate perceived oral health needs among schoolchildren [4,5,10-14].

Apart from estimating individual oral health needs and status, measuring oral impacts in children using the Child-OIDP index is useful in helping clinicians to prioritise treatment provision to those with the highest needs [5,15]. For example, for similar dental conditions, patients with high severity of impact scores might be given treatment priority over those with low impact scores. Subsequently, the index can also be used to evaluate treatment outcomes. Also, it can be used in population surveys to estimate oral health needs, whose information can be used by policy makers to effectively plan, provide and evaluate oral health care services and intervention programmes for the community [2,15].

In Malaysia, there is a need to prioritise oral health promotion programme to schoolchildren with the most oral health needs. Apart from assessing their normative treatment needs, there was also a need to estimate their subjective oral health needs by using a suitable child OHRQoL measure. The chosen OHRQoL index must be easy to use, short, self-administered and relevant to the schoolchildren. It must also possess a scoring method which reflected the frequency and severity of impacts and reliable to be used as a tool for programme evaluation. The Child-OIDP index was selected for the reasons described above. In addition, the index had been developed and tested in an Asian (Thai) population and therefore would be expected to be very similar to the Malaysian setting [4]. Therefore, the objectives of the present study were to develop and test a Malay version of the Child-OIDP index, evaluate its psychometric properties and report on the prevalence of oral impacts on eight daily performances in a sample of 11–12 year old Malaysian schoolchildren.

## Methods

The development of the Malay Child-OIDP index required a cultural adaptation of the English Child-OIDP index into its Malay version. This process involved 2 phases: linguistic and psychometric validations of the Malay Child-OIDP index [16].

Linguistic validation of the Malay Child-OIDP version involved forward translation of the English Child-OIDP index into Malay followed by backward translation of the draft Malay version into English [17]*.* Having obtained permission from the author to adapt the Child-OIDP index, it was first translated into Malay by 3 independent translators who were experts in quality of life measures and proficient in the English language. Then, a meeting with the translators was held to analyse the translations in terms of content and wordings paying attention to conceptual and item equivalence between the original index and its Malay version. Conceptual equivalence refers to whether answers to the same questions reflect the same concept and the concepts are meaningful in both cultures and languages concerned. Item equivalence refers to whether equivalence of meaning of the items is maintained throughout the translation process [18]. Following the meeting, the group agreed on a single consensus translation.

The translated questionnaire was then tested on a non-random sample of 40, 11–12 year old schoolchildren from a school not involved in the final study. The data collection was conducted by the researcher (ZY) and assisted by the classroom teacher. The time taken to answer the questionnaire was noted. Following the test, a discussion with the schoolchildren was held to discuss their understanding of the purpose, content, wording, and general structure of the questionnaire.

The second step involved backward translation of the draft Malay Child-OIDP questionnaire into English. It was carried out by an expert translator from the Department of Asian and European Languages, Faculty of Languages and Linguistics, University of Malaya who was proficient in both English and Malay language. Then, a thorough discussion on the output of the back translation by experts in dental public health, language and quality of life measures was held, comparing the back translation with the original Child-OIDP index. After minor modifications, the back translation of the index was verified by the original authors of the Child-OIDP index at the University College London, UK.

Subsequently, the psychometric properties of the translated index was assessed by testing the index on a non-random sample of 132, 11–12 year old schoolchildren from two urban schools in Kuala Lumpur. One school represented a middle class while the other represented a lower socioeconomic class neighbourhood. All children in Year 6 of both schools were included. The questionnaires were administered in a class room setting. Additional questions on perceived oral health status, satisfaction with oral health, perceived needs for dental treatment and tooth ache experience were given to the schoolchildren. Apart from testing the questionnaire, the feasibility of administrating the instrument under field conditions was also noted. The same questionnaires were administered one week later on 30 of the 132 schoolchildren, representing 22.7% of the sample.

Ethical approval for the study was granted by the ethics committee, Faculty of Dentistry, University of Malaya. Permissions for recruiting the schoolchildren for the linguistic and psychometric validations were obtained from the Ministry of Education Malaysia, district health education authority, school headmasters and parents of the schoolchildren involved.

### Data analysis

The Child-OIDP measures oral impacts on eight daily performances viz: eating, speaking, cleaning teeth, relaxing (including sleep), emotional stability, smiling, doing schoolwork, and socialising. Each impact score was calculated by multiplying the frequency (0 to 3) and severity (0 to 3). Then, the scores of the eight performances were summed up. Finally, the overall score was the sum divided by 72 (maximum possible score) and multiplied with 100 to give a percentage score. As a result, a child can have no oral impact (score = 0) or maximum oral impacts (score = 100) on his eight daily performances. Apart from OIDP scores, Robinson et al. [19] and Gherunpong et al. [5] also suggested an alternative way of quantifying OIDP impacts by calculating the number of OIDP performances with impacts (PWI) affecting a person’s quality of life. Thus, in addition to OIDP scores, the present study also reported findings based on the latter recommendation.

In this study, the psychometric analysis of the Malay OIDP index involved the assessment of internal and test-retest reliability, as well as face, content, criterion and construct validity.

The internal reliability of the Child-OIDP was measured by using standardised Cronhbach alpha coefficient, inter-item correlations and corrected item-total correlations. The test-retest reliability was assessed using weighted kappa for categories of the Child-OIDP scores and the intra-class correlation coefficient (ICC) using two-way random effects model [20]. The reliability tests were carried out to ensure the Child-OIDP index would be interpreted consistently at different times.

*Face and content validity* were tested during the linguistic validation process by experts in dental public health and quality of life measures and through a pilot test involving schoolchildren of similar backgrounds. For criterion validity test, the Malay Child-OIDP index was tested on its ability to measure what it claims to measure [20]. In this study, the Child-OIDP was intended to measure oral impacts which also mirrored levels of oral needs. Consequently, and based on previous study [21], the *criterion validity* of the Malay Child-OIDP was tested by comparing its relationship with perceived need for dental treatment, selected as the proxy measure. *Construct validity* was tested by comparing its relationships with other measures measuring related constructs i.e. perceived satisfaction with mouth, perceived oral health status and toothache experience in the previous 3 months [21,22].

In this study, the Child-OIDP scores were found to be skewed. Therefore, non-parametric statistical tests i.e. Kruskal-Wallis and Mann–Whitney statistics were used to assess relationships between Child-OIDP and subjective measures as mentioned above. The SPSS version 17 was used for analysis. The level for statistical significance was set at p *<* 0.05.

## Results

All 132, 11–12 year old schoolchildren who were selected agreed to participate (response rate 100%). There were slightly more males (n = 72, 55.5%) than females (n = 60, 45.5%). The sample was predominantly Malay (n = 129, 97.7%) with 2 Chinese (1.5%) and 1 Indian (0.8%). The children had varying socioeconomic background.

In terms of face and content validity testing of the Malay Child-OIDP index, it was confirmed by the experts that the Malay Child-OIDP index had achieved conceptual and item equivalence with the original index. Any words which upon translation carried different concept and meaning in the Malay language and culture were replaced with appropriate words with similar concept and meaning to the original item. The face and content validation of the Malay Child-OIDP index was further verified during the pilot study. A discussion with the 40 schoolchildren on the draft Malay Child-OIDP index showed that the questionnaire was well understood in terms of its purpose, content, wording, general layout, instructions and flow with only minor modifications. The time taken to answer the questionnaire was also acceptable i.e. 20–25 minutes. In addition, the feasibility of the instrument administration under field condition had also been verified.

Table 1 shows the inter-item correlation coefficients among the eight item scores of the Child-OIDP index. The correlation coefficients were all positive and ranged from 0.13 (representing relationship between speaking and eating, and speaking and cleaning teeth) to 0.55 (representing relationship between social contacts and doing schoolwork).

**Table 1:** Reliability analysis: inter-item correlation coefficients for the Malay-Child IODP

	**Eating**	**Speaking**	**Cleaning teeth**	**Relaxing**	**Emotional stability**	**Smiling**	**Doing schoolwork**	**Socialising**
Eating	1.00							
Speaking	.13	1.00						
Cleaning teeth	.26	.13	1.00					
Relaxing	.36	.33	.27	1.00				
Emotional stability	.33	.38	.26	.38	1.00			
Smiling	.22	.22	.25	.40	.31	1.00		
Doing schoolwork	.27	.33	.31	.47	.38	.35	1.00	
Socialising	.24	.41	.42	.49	.40	.37	.55	1.00

Table 2 shows the corrected item-total correlation of the eight items. The corrected item-total correlation values were all positive and above 0.3. The standardised Crohnbach’s alpha value was 0.80 and the value did not increase if any of the items were deleted. In terms of test-retest reliability, the weighted kappa value was 0.84 and the intraclass correlation coefficient was 0.79.

**Table 2:** Reliability analysis: corrected item-total correlation of the 8 items of the Malay Child-OIDP and Cronbach’s Alpha coefficients

	**Corrected Item-Total Correlation**	**Cronbach’s Alpha if Item Deleted**
Eating	.39	.78
Speaking	.40	.77
Cleaning teeth	.41	.78
Relaxing	.60	.75
Emotional stability	.54	.75
Smiling	.46	.77
Doing schoolwork	.58	.75
Socialising	.64	.74
Alpha value		0.79
Standardised items alpha		0.80

Table 3 shows the criterion and construct validity testing for associations between Child-OIDP index and subjective oral health measures. The distribution of the Child-OIDP scores was described in quartiles as the scores were skewed. The middle quartile corresponded to the median score. The interquartile range describes the spread of data which is the difference between the lower and the upper quartile. Overall, the median score was higher in children with higher perceived oral health impacts.

**Table 3:** Criterion and construct validity tests for Malay Child-OIDP index: comparison of Child-OIDP scores (0–100) between different categories of subjective outcome variables (N = 132)

**Variables**	**N**	**Mean**	**(SD)**	**Child-OIDP score (Quartiles)**	**P**
Perceived oral health needs^1^					
Yes	60	12.7	(16.8)	(0.3, 8.3, 16.7)	0.002
No	72	5.3	(7.7)	(0.0, 2.8, 8.3)	
Perceived oral health status^2^					
Very good – excellent	83	6.5	(8.2)	(0.0, 5.6, 8.3)	0.036
Moderate – good	45	10.1	(13.4)	(0.0, 8.3, 16.3)	
Poor	4	46.8	(46.8)	(12.5, 27.8, 100)	
Satisfaction with oral health^2^					
Very satisfied	81	5.8	(7.8)	(0.0, 5.6, 8.3)	0.034
Moderate	37	8.2	(11.0)	(0.0, 4.2, 13.9)	
Not satisfied	14	20.2	(27.0)	(4.9, 9.7, 32.0)	
Toothache in past 3 months^1^					
Yes	58	12.5	(16.1)	(1.4, 8.3, 17.0)	
No	74	5.7	(9.4)	(0.0, 1.4, 8.3)	0.001

^1^Mann-Whitney non-parametric statistics.

^2^Kruskal-Wallis non-parametric statistics.

In the study, children with *perceived needs for dental treatmen*t had significantly higher OIDP scores than those who did not have perceived needs for dental treatment (p < 0.05). Schoolchildren who *perceived their oral health status* to be very good to excellent had significantly lower OIDP scores than those who perceived their oral health status to be moderate to good; and those who perceived their oral health status to be poor (p < 0.05). Similarly, children who reported to be very *satisfied with their oral health* had significantly lower OIDP scores than those who reported to be moderately satisfied; and those who reported to be not satisfied with their oral health (p < 0.05). In terms of toothache experience, children who reported to have experienced at least one episode of *toothache in the past 3 months* had significantly higher OIDP scores than those who did not report toothache experience (p = 0.001).

**Figure 1:**
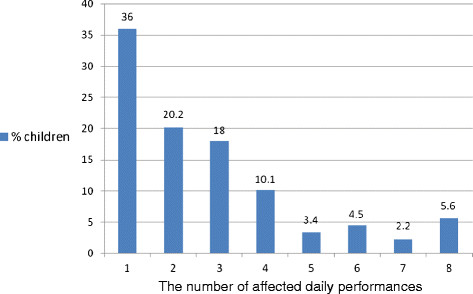
Percentage distribution of the number of OIDP performances affected by oral conditions among individuals with impacts.

Table 4 shows the prevalence of the eight oral impacts on daily performance of the sample. Two-thirds (66.7%) of the sample reported having at least one oral impact affecting their daily performances in the past 3 months. The impacts with the highest reported prevalence were *cleaning teeth* (36.4%), *eating* (34.8%), and *emotional stability* (26.5%). However, in terms of severity of impact, the schoolchildren’s ability to relax was most severely affected by their oral conditions (mean impact score = 27.8, SD = 22.0) followed by their ability to socialise (mean impact score = 26.8, SD = 21.7) and doing schoolwork (mean impact score = 26.3, SD = 22.4). About three-quarters (74.2%) of the children with oral impacts had up to 3 daily performances affected by their oral conditions (Figure 1).

**Table 4:** Prevalence and score of oral impacts on daily performances of the 11–12 year old schoolchildren (n = 132)

**Daily performances**	**N**	**%**	**Mean (SD)**	**Child-OIDP score(Quartiles)**
Any performance affected	88	66.7	13.2 (14.4)	(5.6, 8.3, 16.7)
Cleaning teeth	48	36.4	16.4 (17.4)	(6.9, 11.1, 18.4)
Eating	46	34.8	17.0 (17.8)	(5.6, 11.1, 22.6)
Emotional stability	35	26.5	21.5 (18.9)	(8.3, 16.7, 27.8)
Smiling	32	24.2	19.6 (19.8)	(8.3, 11.8, 24.6)
Speaking	25	20.0	21.7 (21.6)	(8.3, 13.9, 32.6)
Relaxing	21	16.0	27.8 (22.0)	(12.5, 24.3, 39.6)
Doing schoolwork	20	15.2	26.3 (22.4)	(11.1, 18.8, 39.2)
Socialising	19	14.4	26.8 (21.7)	(13.9, 19.4, 36.1)

## Discussion

In the present study, the development of the Malay version of the Child-OIDP was carried out in accordance with standard protocols [17] and following previous related studies [10,11,14,21,23]. The forward and backward translations of the Child-OIDP were done by local experts in dental public health and quality of life and also proficient in English and Malay languages. It was confirmed that the backward translation of the Malay version into English had equivalent subject content and meaning and had closely similar wordings to the original Child-OIDP, taking into account minor modifications to the Malay version based on feedbacks from the pilot study. This backward translation was verified by the original developers of the Child-OIDP instrument after addressing a few minor queries. In this study, the Malay version of the Child-OIDP index had been proven to be valid and reliable for use among 11–12 year old urban schoolchildren in Malaysia. Its psychometric properties in terms of face and content validity, criterion and construct validity as well as internal and test-retest reliability had been successfully tested and empirically verified.

In the reliability test analysis, the inter-item correlation coefficients of the eight item scores were all positive, above zero and between 0.13 to 0.55. The correlation coefficient values were not too large for any item to be deemed redundant. This indicates that the Malay Child-OIDP index items are well correlated with one another in a positive manner and are relevant to constitute an index. The corrected item-total correlation of the eight items was above the recommended level of 0.2 indicating that all items correlated well with total score and the scale overall [20]. In addition, the value for standardised items Cronbach alpha was 0.80 indicating the index was highly reliable to be used in the Malaysian setting [24]. The Cronbach alpha coefficient value did not increase when any of the items were deleted indicating no item should be removed from the index as all items were highly correlated and relevant. In terms of the test-retest reliability of the Malay Child-OIDP, the index had been proven to be reliable in yielding consistent scores. The weighted kappa and the intra-class correlation coefficients were excellent with values very near to or above 0.8.

During the pilot study, it was noted that a small number of schoolchildren had difficulty to identify oral episodes in the past 3 months. Most of the uncertainties rallied around episodes which had occurred ‘some time ago’ but less than 6 months. As a result, a few schoolchildren required a relatively longer time to complete the first part of the questionnaire despite having written the names of the 3 previous months for guidance. Following discussion, a solution was proposed by the students to refer the oral episodes to a significant social event that took place at the school approximately 3 months ago. First, the previous third month was identified. Then a significant event in that month was recorded based on the school calendar. Then all oral episodes which had occurred on or after the significant event would be reported. This proved to be very helpful and led to minor modifications to the questionnaire instruction. This experience showed that, in addition to written instructions, a few children may need help through verbal instructions when answering the questions. Thus it is recommended that future studies using the index should involve the researcher’s presence when administrating the questionnaire.

The sample size (n = 132) may be criticised to be a bit small but according to Clark and Watson, the sample size to be used in psychometric test of an index depends on the number of items in the index. In general, for index with 20 items or less, a sample size between 100 to 200 subjects was reasonable [25]. Thus, for the Malay Child-OIDP index with eight items, the sample size of 132 subjects was deemed sufficient for the purpose of testing the psychometric properties of the Malay Child-OIDP index. Also, for the same purpose, the use of non-random sample was justified as long as the sample is relevant. In our study, the index was culturally adapted for use among 11–12 year olds, and thus was tested on similar aged children.

In terms of the overall prevalence of oral impacts on daily performances of the schoolchildren, two-thirds (66.7%) reported having at least one performance affected by oral conditions. This prevalence was higher than findings on a similar age group in London, Tanzania and Sudan, [11,13,26] but lower than those in Brazil, Thailand and France [5,10,14]. In the present study, the most affected performance was *cleaning teeth,* followed by *eatingemotional stabilitysmiling* and *speaking*. The two least prevalent impacts were doing *schoolwork* and *socialising*. However, it was interesting to note that the opposite trend was true in terms of severity of impact. The ability to *relax well, socialise* and *doing schoolwork* were perceived to be most severely affected by oral conditions. This indicates that schoolchildren at this age value certain activities more than others. Doing schoolwork, socialising with friends and relaxing are activities which are important to schoolchildren. In the sample, disturbances in these three activities were highly significant to them as it concerned their performance at school and socialising with friends. Also, about three in every four (74.2%) schoolchildren with oral impacts had up to three daily performances affected by their oral conditions. This further indicates that oral conditions do impact on the schoolchildren’s daily performance, quality of life and well-being.

This study had a few limitations. Although the sample of schoolchildren came from varying academic abilities, socioeconomic background and the gender balance was acceptable, this study was conducted in an urban area. Thus, further studies to evaluate the validity and reliability of the Child-OIDP on rural schoolchildren are recommended. Also, a bigger sample is recommended to confirm the prevalence and severity of oral impacts among Malaysian schoolchildren. Another limitation was that the Child-OIDP was not tested against any clinical measure of oral health. This should be performed in further studies.

## Conclusions

This study indicates that the Malay Child-OIDP index is a valid and reliable measure to be used as an OHRQoL index among 11–12 year old urban schoolchildren in Malaysia.

## Abbreviations

Child-OIDP: Child Oral Impacts on Daily Performance; OHRQoL: Oral Health Related Quality of Life; PWI: Performance with impact; ICC: Intraclass correlation coefficient.

## Competing interests

The authors declare that they have no competing interests.

## Authors’ contributions

ZYMY contributed in the design of the study, development of the Malay Child-OIDP index, acquisition of data, analysis and interpretation of data, and drafting the manuscript. NJ advised on the study design, development of the Malay Child-OIDP index and revising the manuscript. Both authors read and approved the final manuscript.
